# Rotavirus Vaccine Administration in Preterm and Medically Fragile Infants Admitted to Neonatal Intensive Care Units: Second Phase Enrollments and Final Results of a Multicenter Observational Study Conducted in Sicily, Italy

**DOI:** 10.3390/vaccines13020100

**Published:** 2025-01-21

**Authors:** Claudio Costantino, Nicole Bonaccorso, Walter Mazzucco, Francesco Balsamo, Martina Sciortino, Mario Palermo, Kim Maiolo, Lucia Gabriella Tina, Pasqua Maria Betta, Mariacarmela Caracciolo, Carmine Mattia Loretta, Domenico Cipolla, Salvino Marcello Vitaliti, Domenica Mancuso, Giuliana Vitaliti, Vincenzo Rosella, Giuseppa Pinello, Fabio Tramuto, Emanuele Amodio, Francesco Vitale

**Affiliations:** 1 Department of Health Promotion Sciences, Maternal and Infant Care, Internal Medicine and Excellence Specialties (PROMISE) “G. D’Alessandro”, University of Palermo, 90127 Palermo, Italy; claudio.costantino01@unipa.it (C.C.); walter.mazzucco@unipa.it (W.M.); francesco.balsamo02@unipa.it (F.B.); martina.sciortino@unipa.it (M.S.); fabio.tramuto@unipa.it (F.T.); emanuele.amodio@unipa.it (E.A.); francesco.vitale@unipa.it (F.V.); 2 PhD National Programme in One Health Approaches to Infectious Diseases and Life Science Research, Department of Public Health, Experimental and Forensice Medicine, University of Pavia, 27100 Pavia, Italy; 3 Regional Health Authority of Sicily, Via Vaccaro 5, 90145 Palermo, Italy; servizio4.dasoe@regione.sicilia.it; 4 Neonatal Intensive Care Unit, Garibaldi Hospital, 95124 Catania, Italy; kimmaiolo@hotmail.com (K.M.); gabriella.tina@tiscali.it (L.G.T.); 5 Neonatal Intensive Care Unit, University Hospital of Catania (G. Rodolico), 90123 Catania, Italy; mlbetta@yahoo.it (P.M.B.); melacaracciolo@virgilio.it (M.C.); lorettamattia@hotmail.com (C.M.L.); 6 Department of Pediatric Emergency, ARNAS Civico, Di Cristina Benfratelli, 90127 Palermo, Italy; 7 Neonatology Unit, NICU and Creche, ARNAS Civico, 90127 Palermo, Italy; marcello.vitaliti@gmail.com (S.M.V.); domenica.mancuso@libero.it (D.M.); giulianavitaliti@gmail.com (G.V.); 8 Neonatal Intensive Care Unit, Maternal and Child Department, Buccheri La Ferla Fatebenefratelli Hospital, 90123 Palermo, Italy; vincenzorosella@libero.it (V.R.); giusypinello@gmail.com (G.P.)

**Keywords:** rotavirus, rotavirus gastroenteritis, hospitalizations, rotavirus vaccination, preterm infants

## Abstract

Rotavirus disease is the world’s leading cause of severe gastroenteritis and mortality in children up to 5 years of age. Premature infants are more vulnerable to rotavirus gastroenteritis (RVGE) and its complications. International authorities strongly recommend vaccination because of the consistent reduction in infections, hospitalizations, deaths, and related costs. Background/Objectives: The objective of the present study was to evaluate the safety of anti-rotavirus vaccination in premature infants admitted and vaccinated in the main Sicilian Neonatal Intensive Care Units (NICUs) during the pandemic period. Methods: The human monovalent rotavirus vaccination (RV1) was administered to preterm infants of gestational age ≥28 weeks in the main Sicilian NICUs from January 2020 to December 2022, as a prolongation of a similar study conducted from April 2018 to December 2019. Rotavirus vaccinations were provided both to hospitalized infants and to those returning for post-discharge follow-up, beginning at six weeks of age according to the official immunization schedule. All potential adverse events—whether expected, unexpected, or serious—were recorded from the day of vaccination through 14 days (first follow-up) and 28 days (second follow-up) after each of the two scheduled doses. Results: A total of 355 preterm infants were fully vaccinated with RV in four Sicilian NICUs. The mean gestational age of newborns was 33.2 weeks (±2.7), 53% of whom were male. Vaccination was performed on average at 7 weeks of age (±2.1), and the mean weight at the time of vaccination was 3439 g (SD ± 745.2). No expected/unexpected or serious adverse events were observed either within the 14-day or within the 28-day period after administration of both two doses. Conclusions: Data confirm that vaccination in preterm infants ≥28 weeks gestational age is safe. The prolongation of this Public Health strategy, strongly recommended by the Sicilian Health Department during the pandemic period that also generally has led to a reduction of vaccination adherence and acceptance of pediatric vaccination, demonstrates the importance of multidisciplinary collaboration with neonatologists and pediatricians to continue promoting in-hospital vaccinations for fragile subjects.

## 1. Introduction

Globally, rotavirus remains the most common cause of severe gastroenteritis and a key contributor to mortality among children under five years of age [1,2,3,4]. Before the introduction of the rotavirus vaccine (RV), rotavirus accounted for approximately 3.6 million cases of rotavirus gastroenteritis (RVGE) annually in children aged 0 to 59 months, leading to 87,000 hospitalizations (representing 14–54% of all hospitalizations due to acute gastroenteritis) and approximately 700,000 medical visits in Europe [5,6].

International health authorities strongly advocate rotavirus vaccination, recognizing it as the most effective measure to curtail RVGE in young children [7]. Two vaccines are available for rotavirus protection: RV5, a pentavalent human–bovine reassortant live oral vaccine, administered in a 3-dose series, and RV1, a monovalent attenuated human live oral vaccine, administered in a 2-dose series. Both vaccines are safe and effective [8,9]. RV5 and RV1 can be given to infants born after at least 28 weeks of gestation, who are not immunocompromised, starting from 6 weeks of age (42 days), with a minimum interval of 4 weeks between doses [10].

Multiple studies have shown that the benefits of vaccination far outweigh the modest risk of intussusception, which can be a potential cause of hesitancy regarding RV adherence [11,12,13].

Premature infants can be particularly vulnerable to severe gastrointestinal disease due to various factors. In particular, delayed enteral feeding and frequent use of histamine-2 receptor antagonists or proton pump inhibitors reduce gastric acidity, an essential component of the innate immune defense against rotavirus infection [13,14]. Once infected, preterm infants experience a more severe form of RVGE compared to full-term infants, with increased risk of complications, such as seizures [15,16].

Preterm infants are particularly vulnerable to severe rotavirus infections, which can present with increased bowel distension, abdominal distension, mucoid stools, necrotizing enterocolitis, and secondary bacteremia, leading to higher hospitalization rates [16,17,18,19]. Very low birth weight infants are 2.6 times more likely to be hospitalized compared to their full-term peers [20].

Additionally, preterm infants have lower maternal antibody levels due to limited placental transfer of immunoglobulin G (IgG) before 28 weeks of gestation. Prolonged hospital stays, multiple comorbidities, and low birth weight further increase the risk of nosocomial infections [21,22,23]. For this group, rotavirus vaccination should be a top public health priority [24,25,26].

Despite the higher severity of rotavirus disease in early infancy, rotavirus vaccination is not routinely administered to infants in Neonatal Intensive Care Units (NICUs) in Italy and several other European countries [27], even though multiple studies have confirmed the efficacy and safety of rotavirus and other vaccines in preterm infants [28,29,30].

Numerous studies in both the United States and Europe have shown that routine vaccinations, including live-attenuated vaccines, are generally as safe and well tolerated in preterm infants as they are in full-term infants [31,32,33].

This study presents the results of a multicenter investigation carried out in four major Neonatal Intensive Care Units (NICUs) in the Sicilian region during the pandemic period (2020–2022). Its primary objective is to assess potential adverse events (expected, unexpected, and serious) associated with the human rotavirus vaccine (RV1) in preterm and medically fragile infants. It serves as a continuation of a previously published multicenter study on the safety of rotavirus vaccination in preterm and medically fragile infants admitted to NICUs between April 2018 and December 2019 [28].

The primary aim of this analysis is to provide additional data on the safety of rotavirus vaccination in such a vulnerable population, including preterm and fragile infants, and to reaffirm the safety of this crucial public health measure, which helps prevent RVGE-related hospitalizations, complications, deaths, and healthcare costs.

## 2. Materials and Methods

This study is a prolongation of a previous observational multicenter study conducted in the six largest NICUs in Sicily (three in Palermo—PA, two in Catania—CT, and one in Messina—ME) from April 2018 to December 2019, which enrolled 449 infants [28].

Vaccine administration and data collection were hindered by the COVID-19 pandemic, which significantly disrupted activities in both the general population and hospital settings. Despite these challenges, four out of six Sicilian NICUs continued, through several difficulties, enrollments, and data collection, adhering to the previous research protocols.

During the COVID-19 pandemic (1 January 2020 to 31 December 2022), we carried out an observational, descriptive, non-controlled, and non-randomized study in four of the region’s largest NICUs to assess the safety of RV1 vaccination administered in these units. Specifically, enrollment took place at the “Buccheri La Ferla Fatebenefratelli” hospital in Palermo, the “Civico-Di Cristina-Benfratelli” hospital in Palermo, the “New Garibaldi Hospital-Nesima” in Catania, and the “University Hospital Gaspare Rodolico” hospital in Catania, operating in the two most populous LHAs of Catania and Palermo that accounted more than 50% of the Sicilian general population.

Sicily, Italy’s fourth most populous region, with 4,814,016 inhabitants and nearly 35,000 deliveries per year [34,35], has 47 public or private accredited clinics where deliveries are performed. Among the hospitals involved in the study, the “Buccheri La Ferla Fatebenefratelli” hospital in Palermo ranks first in delivery volume (2201 deliveries), followed by the “Garibaldi-Nesima” hospital in Catania (2142 deliveries), the “Civico-Di Cristina-Benfratelli” hospital in Palermo (1980 deliveries), and the University Hospital in Catania (1347 deliveries) [36].

Furthermore, each of the seven remaining Local Health Authorities (LHAs) in Sicily—Agrigento, Caltanissetta, Enna, Messina, Ragusa, Siracusa, and Trapani—has its own NICU (three in the Messina LHAs). Furthermore, there are no private NICUs in Sicily, and very preterm or medically fragile newborns born in private clinics are always transferred to one of the public NICUs, ensuring access to the required specialized care.

In the previous phase of the study, two other university hospitals, one in Palermo and one in Messina, were also involved; however, during the COVID-19 pandemic, these hospitals continued RV1 vaccination for preterm infants but not active surveillance on safety of RV1 vaccination, as requested by the study protocol.

This current phase of the study, therefore, focuses on the four NICUs that continued their full participation in the study protocol, despite the challenges posed by the ongoing pandemic.

The scientific coordination of this project was carried out by the Department of Health Promotion, Maternal and Infant Care, Internal Medicine, and Excellence Specialties (PROMISE) at the University of Palermo, in collaboration with the Regional Health Department of the Sicilian Region. To ensure our sample was large enough to detect adverse events (AEs) with acceptable precision, we performed a single-proportion sample size calculation based on an expected AE incidence of 2%, a margin of error (d) of ±2%, and a confidence level of 99%. Using the standard formula for a single proportion, we estimated that approximately 326 participants would be required. Sicily was the first Italian region to adopt universal rotavirus vaccination (URV) in its immunization schedule, implementing it in January 2013 [37]. Subsequently, in 2017, Italy introduced a nationwide URV program through its National Vaccination Plan (2017–2021) [38]. Under the regional plan, the monovalent rotavirus vaccine (RV1) was chosen for its well-documented safety and efficacy profile. This live-attenuated vaccine, derived from a human rotavirus strain, is administered orally in two doses, at least four weeks apart, beginning at six weeks of age and completing by 24 weeks of age. In accordance with the Summary of Product Characteristics (SmPC), RV1 can be given to preterm infants of at least 28 weeks gestational age, starting from six weeks of life [39]. The study protocol excluded infants with unstable clinical conditions—such as those requiring cardiorespiratory support, parenteral nutrition, or antibiotic therapy for an active infection—as well as those with a history of intussusception, necrotizing enterocolitis (NEC), intestinal malformations, or immunodeficiency.

**Vaccine Administration and Adverse Event Monitoring**

Vaccine doses were administered both to inpatients and to outpatients returning for post-discharge follow-up at the participating NICUs [39]. Possible adverse events (expected, unexpected, or serious) were tracked from the day of vaccination up to 14 days (first evaluation) and 28 days (second evaluation) after each of the two scheduled doses. Following Directive 2001/20/EC Art. 2 [40], adverse events were categorized as follows:

- *Expected adverse event (**EAE)***: a reaction listed in the vaccine’s data sheet.
- *Serious unexpected adverse event (uAE)*: a serious adverse reaction whose nature, severity, or outcome does not align with the reference safety information.
- *Serious adverse event (SAE)*: any harmful clinical event that, regardless of the dose, requires hospitalization or prolongs ongoing hospitalization, results in severe or prolonged disability or incapacity, causes a congenital anomaly or birth defect, is life-threatening, or results in death.

Parents took part in active surveillance by completing a diary card or reporting any symptoms via telephone. In the case of hospitalization, surveillance was directly managed by healthcare personnel. Parents documented whether symptoms were absent, mild (no pediatric consultation), moderate (pediatric consultation needed), or severe (hospitalization required), indicating the onset and resolution dates. Informational materials were provided to help parents recognize potential adverse events, such as diarrhea, urticaria, and intussusception, along with a thermometer for daily temperature checks. Aspects like abdominal discomfort, irritability, and colic were reported subjectively based on parental observation.

According to the SmPC, adverse events of particular interest were

1. **Common events**: Diarrhea, irritability;
2. **Uncommon events**: Abdominal pain, flatulence, skin irritation;
3. **Very rare events**: Urticaria, intussusception, visible blood in the stool, and (in very preterm infants) prolonged apnea.

This study received prior approval from the Ethical Committee of the University Hospital of Messina (protocol no. 526, 6 April 2018).

### Statistical Analysis

Absolute and relative frequencies were calculated for the categorical (qualitative) variables. Quantitative data were summarized as mean (±standard deviation, DS) if normally distributed or median (interquartile range, IQR) elsewhere. All data were entered into a database created with EpiInfo 3.5.4 (Centers for Disease Control and Prevention, Atlanta, GA, USA). All data were analyzed using the statistical software package Stata/MP 14.1 (StataCorp LP, College Station, TX, USA). The adverse event rate was calculated as the overall number of adverse events occurring within 28 days after any dose, per number of doses administered.

## 3. Results

A total of 355 preterm or fragile infants (out of 523 potentially eligible newborns, due to some parents declining consent; adherence rate = 67.8%) were enrolled and fully vaccinated (completion of RV1 two-dose cycle) between 1 January 2020 and 31 December 2022. In order to assess sample size representativeness, we estimated that approximately 326 participants would be required, based on an expected adverse event rate of 2%, a desired precision of ±2%, and a 99% confidence interval. Table 1 presents the main characteristics of the preterm infants enrolled at the time of the first vaccine administration and followed through to the completion of the vaccine cycle.

Of the enrolled infants, 188 (53.0%) were male and 167 (47.0%) were female, with a mean weight of 3439.2 g (±745.2) at the time of the first dose. Most infants were formula-fed (202; 68.2%), while 20 (9.5%) were exclusively breastfed, and 66 (22.3%) were both breastfed and formula-fed.

The most common comorbidities at birth were moderate preterm birth in 198 infants (53.7%), very preterm birth (gestational age <32 weeks) in 69 infants (23.4%), and twin birth in 108 cases (30.6%). Additionally, 31 infants (8.7%) were born with respiratory distress syndrome (RDS), 29 (8.2%) had hyperbilirubinemia, 28 (7.9%) had hypoglycemia, and 24 (6.8%) had hypocalcemia. The mean gestational age at birth was 33.2 weeks (±2.7), and the first dose of the RV1 vaccine was administered at an average age of 54.9 days (±14.9).

Overall, 5.4% of preterm infants received their first vaccination while still in hospital wards or neonatal intensive care units, whereas the remaining vaccinations were administered during outpatient visits, either as part of routine biweekly checkups or at territorial clinics run by local health authorities (LHAs). The second dose of RV1 was given at an average age of 92.0 days (±21.1), with an average weight of 4225.7 g (±722.1) at the time of administration.

As shown in Table 2, no expected, unexpected, or serious adverse events were reported within either the 14-day or 28-day periods following the administration of each of the two doses.

Table 3 categorizes preterm infants by gestational age and the number of days completed at the time of the first administration. These details were not analyzed in the previously published study, so this table provides a comprehensive summary of data for all preterm or fragile infants enrolled in the study since its beginning in April 2018.

Overall, 425 infants (52.9%) were vaccinated between the 42nd and 50th day of life, 246 (30.6%) between the 51st and 61st day, and the remaining 133 (16.5%) after the 61st day. Most infants—444 (55.3%)—were categorized as Late Preterm, followed by 173 (21.4%) categorized as Low Preterm, 157 (19.6%) as Moderate Preterm, and 30 (3.7%) as Full-Term.

Among the 425 infants vaccinated between the 42nd and 50th day, 262 (32.6%) were Late Preterm, 84 (10.5%) were Moderate Preterm, 61 (7.5%) were Low Preterm, and 18 (2.3%) were Full-Term. Of the 246 vaccinated between the 51st and 61st day, 136 (16.9%) were Late Preterm, 52 (6.5%) were Moderate Preterm, 51 (6.4%) were Low Preterm, and 7 (0.8%) were Full-Term. Among the 133 vaccinated after the 61st day, 61 (7.5%) were Low Preterm, 46 (5.8%) were Late Preterm, 21 (2.6%) were Moderate Preterm, and 5 (0.6%) were Full-Term.

Finally, the overall rate of expected adverse events (EAEs) was 68.4 per 100,000 doses administered, with results lower among Low Preterm (57.8 per 100,000) and Moderate Preterm (31.8 per 100,000) infants vaccinated (Table 3).

## 4. Discussion

This study carried out a safety analysis of the RV1 vaccine using an active surveillance system, evaluating the incidence of adverse events from the day of vaccination through 14 days (first follow-up) and 28 days (second follow-up) after each of the two scheduled doses, in a subgroup of preterm and fragile newborns. Among the 355 preterm infants vaccinated between 2020 and 2022, no adverse events were observed during the 14- or 28-day periods after the administration of either dose. Also, the overall rate of EAEs diminished significantly from that observed in the pre-COVID-19 study (161.8 per 100,000), reaching an overall rate of 68.4 per 100,000 doses administered [28]. Our findings, especially overall rate observed, align with several other investigations assessing adverse events in preterm and medically fragile infants receiving the monovalent rotavirus vaccine (RV1). For instance, Marcinek et al. observed no adverse effects in a cohort of 126 preterm infants receiving rotavirus vaccination [29], while Roué et al. similarly reported that vaccine safety in premature infants was comparable to that in term infants, suggesting similar tolerance across these populations [41]. Taken together, these studies reinforce the conclusion that rotavirus vaccination does not appear to pose additional safety concerns, even in a subgroup of infants who are both younger (adjusted gestational age) and medically fragile.

Because of the heightened vulnerability of preterm infants to rotavirus (RV) infection and the demonstrated effectiveness of RV vaccines, health authorities advise vaccination for all preterm neonates with stable clinical conditions, regardless of gestational age [42,43]. Nevertheless, the practice of administering the first dose in the Neonatal Intensive Care Unit (NICU) remains infrequent, contributing to potential delays or omissions in immunization. In our study, only a small portion of preterm infants received the first dose of the rotavirus vaccine during their stay in the NICU, while the majority were vaccinated post-discharge. This low percentage may be due to concerns regarding potential horizontal transmission of the vaccine virus in this setting. To improve vaccination rates, it is essential to raise awareness among neonatologists about the safety and effectiveness of the rotavirus vaccine (RV) [33,44,45].

Nosocomial rotavirus (nRV) disease has been highlighted by some authors as an emerging public health issue [46]. Before the introduction of RV vaccination, the peak of hospitalizations due to rotavirus gastroenteritis in late winter and early spring coincided with a surge in infections caused by other respiratory viruses, such as respiratory syncytial virus, influenza, and adenovirus. This confluence of infections led to overcrowded pediatric wards and heightened the risk of nosocomial transmission, including rotavirus [47]. A review by STIKO suggested that RV vaccination can help prevent in-hospital cases of rotavirus gastroenteritis [48]. Rotavirus is highly transmissible due to several factors: the high number of viral particles excreted during the acute phase, its low infectious dose (<100 viral particles), prolonged environmental persistence, and relative resistance to disinfectants [46].

Preterm infants hospitalized from birth face an elevated risk of hospital-acquired rotavirus infections compared to term infants, highlighting the need to administer the vaccine both during the NICU stay and during follow-up visits, in line with standard immunization guidelines [49,50]. Concerns have been raised about the possibility of vaccine virus transmission to other preterm infants in the NICU, yet this risk is primarily associated with the first dose. Notably, very few preterm infants remain hospitalized beyond 16 weeks of age, the upper limit for receiving the second dose. Furthermore, while some studies have examined the risk of viral shedding after RV1 and RV5 vaccination in full-term infants, the results have varied based on the detection methods used, suggesting that the actual risk of transmission in the NICU may be lower than previously thought [17,18,50].

A retrospective study by Monk et al. evaluated gastrointestinal symptoms in 96 vaccinated and 801 unvaccinated preterm infants within 15 days of receiving the RV5 vaccine in the NICU between 2008 and 2010. The study, which involved preterm infants who were able to tolerate some enteral feeding, found no symptoms attributable to RV5 in the vaccinated group and no evidence of transmission to unvaccinated infants [51]. Similarly, research by Sicard et al. demonstrated that vaccine-derived virus shedding occurs in almost all infants, particularly during the first week after the initial dose, but transmission has rarely been documented, limited in home environments, and no cases of transmission were observed in the NICU setting [52].

Despite the vulnerability of the population enrolled in the Sicilian NICUs, this study observed a zero rate of adverse events (AEs) when compared to data from the Italian pharmacovigilance surveillance system [53]. Multiple scientific committees across the United States, Germany, Spain, and Sweden recommend that preterm infants receive RV immunization, including during their hospital stay [54]. In particular, the American Committee on Immunization Practices (ACIP) stresses that the potential benefits of rotavirus vaccination for preterm infants (born at <37 weeks gestation) exceed the risks, endorsing the same schedule as for term infants once their clinical status is stable. Moreover, the ACIP encourages immunization either at NICU discharge or afterward, stating that the benefits outweigh the potential risks of viral spread in inpatient or nursery settings [55].

Various scientific societies, including the Standing Committee on Vaccination (STIKO) in Germany, the Spanish Pediatric Association, and the Swedish Public Health Agency, recommend the vaccination of preterm infants even during hospitalization [26,56,57]. STIKO emphasizes that the benefits of RV vaccination in NICUs, particularly in preventing nosocomial RV infections, far outweigh the minimal risk of transmission to other hospitalized patients. The Spanish Pediatric Association updated its recommendations in 2023, advocating for vaccination between 6 and 12 weeks after birth, while the Swedish Public Health Agency suggests that preterm infants receive their first dose of the RV vaccine during hospitalization, followed by subsequent doses according to the standard vaccination schedule [26,56,57].

In this study, most of the preterm infants (52.9%) were vaccinated between the 42nd and 50th day of life, reflecting a high level of awareness regarding the potential risks associated with delaying vaccination. Otherwise, in our study, lower vaccination rates among Low Preterm infants may be due to the need for a longer period to achieve clinical stability (7.5%). Nonetheless, it is reassuring that, when possible, these infants were vaccinated as early as feasible, in accordance with the vaccine’s data sheet, highlighting the commitment to protecting this fragile population [28,58].

There are some limitations to this study. Adverse event reporting relied on parents, and this approach could introduce bias resulting in underreporting. Parents may miss subtle symptoms, misinterpret normal behavior as adverse effects, or hesitate to report mild symptoms they consider unimportant. Additionally, while the questionnaires were administered during active surveillance (either in-person or via phone calls), which minimizes the likelihood of recall bias, reporting bias cannot be completely excluded.

Another important limitation is the absence of a control group. This limits the ability to directly compare the frequency of adverse events with that of a non-vaccinated population, making it more challenging to rule out the possibility that some adverse events could have occurred regardless of the vaccination. However, the lack of adverse drug reactions (ADRs) observed in this study partially mitigates this limitation, as there were no adverse effects to compare or attribute specifically to the intervention. Lastly, the possibility of vaccine transmission within the NICU was not evaluated, as fecal samples were not collected or tested. Nonetheless, existing literature suggests that vaccine shedding is limited and that transmission of vaccine-derived rotavirus strains between vaccinated and unvaccinated infants in NICUs is unlikely. No vaccine virus genome has been detected in fecal samples from unvaccinated infants, in contrast to those from vaccinated infants [59].

## 5. Conclusions

These findings confirm the absence of expected, unexpected, and serious adverse events, reinforcing the favorable safety profile of the monovalent rotavirus vaccine for preterm infants born at ≥28 weeks gestation. Due to the multiple comorbidities often present in these extremely fragile infants, prolonged hospital stays are frequently required, which can lead to missed opportunities for timely vaccination and thus an increased risk of contracting rotavirus infection also during the hospitalization. Ensuring a consistent approach to vaccination across all Sicilian and Italian Neonatal Intensive Care Units (NICUs)—through the collaboration of neonatologists, other healthcare professionals, and families—could substantially enhance protection for these vulnerable newborns, who are at greater risk of severe rotavirus gastroenteritis and nosocomial infection. In conclusion, these findings reinforce confidence in the rotavirus vaccine, facilitating a calm and informed decision-making process for NICU healthcare workers and families alike. Additionally, the results provide a strong foundation for improving vaccine coverage both regionally in Sicily and at the national level in Italy.

## Figures and Tables

**Table 1:** Characteristics of preterm newborns enrolled at first and second rotavirus vaccination dose (n = 355, enrollment period 2020–2022).

	n (%)
**Sex, n (%)**	
-Male	188 (53.0)
-Female	167 (47.0)
**Average gestational age (weeks) ± SD**	**33.2** **± 2.7**
**Average weight at first vaccination dose, grams ± SD**	**3439.2 ± 745.2**
**Feeding, n (%)**	
-Breast-feeding	20 (9.5)
-Artificial feeding	202 (68.2)
-Mixed feeding	66 (22.3)
**Average number of days completed at second vaccination dose ± SD**	**92.0 ± 21.1**
**Average number of weeks completed at second vaccination dose ± SD**	**13.1 ± 3.0**
**Comorbidities at birth, n (%)**	
-Moderate preterm birth (32 to 37 weeks)	198 (55.8)
-Very preterm birth (<32 weeks)	69 (19.4)
-Twinning	108 (30.4)
-Respiratory distress syndrome (RDS)	31 (8.7)
-Hypoglycaemia	28 (7.9)
-Hypocalcaemia	24 (6.8)
- Hyperbilirubinemia	29 (8.2)

**Table 2:** Adverse events recorded 14 and 28 days following the first and second doses of the monovalent rotavirus vaccine (RV1) in 355 infants, enrolled from 2020 to 2022. EAEs = expected adverse events; uAEs = unexpected adverse events.

	Fever (≥38.5 °C) n (%)	Abdominal Colic n (%)	Diarrhoea n (%)	Vomiting n (%)	Intestinal Invagination n (%)	Food Refusal n (%)	uAEs n (%)
**EAEs 14 days after first/second administration**	0	0	0	0	0	0	0
**EAEs 28 days after first/second administration**	0	0	0	0	0	0	0

**Table 3:** Classification of enrolled infants vaccinated at the main Sicilian NICUs and overall number of EAEs observed, in accordance with gestational age and days completed at the time of first administration, since the beginning of the project in April 2018 (n = 804).

	Classification According to Gestational Age					
		Low Preterm (28 Weeks to 31 Weeks + 6 Days) n (%)	Moderate Preterm (32 Weeks to 33 Weeks + 6 Days) n (%)	Late Preterm (34 Weeks to 36 Weeks + 6 Days) n (%)	Full-Term New Born (37 Weeks to 40 Weeks + 6 Days) n (%)	Tot. n (%)
*Days at 1st dose*	**42°–50°**	61 (7.5%)	84 (10.5%)	262 (32.6%)	18 (2.3%)	**425** **(52.9%)**
	**51°–61°**	51 (6.4%)	52 (6.5%)	136 (16.9%)	7 (0.8%)	**246** **(30.6%)**
	**>61°**	61 (7.5%)	21 (2.6%)	46 (5.8%)	5 (0.6%)	**133** **(16.5%)**
	**Tot.** **n (%)**	**173** **(21.4%)**	**157** **(19.6%)**	**444** **(55.3%)**	**30** **(3.7%)**	**804** **(100%)**
*Overall EAEs within 28 days after first or second dose* *(rate per 100,000 doses administered)*		2 (57.8 per 100,000)	1 (31.8 per 100,000)	7 (78.8 per 100,000)	1 (166.7 per 100,000)	**11** **(68.4 per 100,000)**

## Data Availability

Data obtained in the present study are available upon request to the corresponding author.
